# Transcriptome Profiling of Mouse Corpus Callosum After Cerebral Hypoperfusion

**DOI:** 10.3389/fcell.2021.685261

**Published:** 2021-06-17

**Authors:** Hajime Takase, Gen Hamanaka, Ryo Ohtomo, Hidehiro Ishikawa, Kelly K. Chung, Emiri T. Mandeville, Josephine Lok, Myriam Fornage, Karl Herrup, Kai-Hei Tse, Eng H. Lo, Ken Arai

**Affiliations:** ^1^Neuroprotection Research Laboratory, Department of Radiology and Neurology, Massachusetts General Hospital, Harvard Medical School, Charlestown, MA, United States; ^2^Institute of Molecular Medicine, McGovern Medical School, The University of Texas Health Science Center at Houston, Houston, TX, United States; ^3^Human Genetics Center, Division of Epidemiology, School of Public Health, University of Texas Health Science Center at Houston, Houston, TX, United States; ^4^Department of Neurobiology and ADRC, University of Pittsburgh, Pittsburgh, PA, United States; ^5^Department of Health Technology and Informatics, The Hong Kong Polytechnic University, Kowloon, Hong Kong

**Keywords:** cerebral hypoperfusion, corpus callosum, dementia, white matter, RNAseq

## Abstract

White matter damage caused by cerebral hypoperfusion is a major hallmark of subcortical ischemic vascular dementia (SIVD), which is the most common subtype of vascular cognitive impairment and dementia (VCID) syndrome. In an aging society, the number of SIVD patients is expected to increase; however, effective therapies have yet to be developed. To understand the pathological mechanisms, we analyzed the profiles of the cells of the corpus callosum after cerebral hypoperfusion in a preclinical SIVD model. We prepared cerebral hypoperfused mice by subjecting 2-month old male C57BL/6J mice to bilateral carotid artery stenosis (BCAS) operation. BCAS-hypoperfusion mice exhibited cognitive deficits at 4 weeks after cerebral hypoperfusion, assessed by novel object recognition test. RNA samples from the corpus callosum region of sham- or BCAS-operated mice were then processed using RNA sequencing. A gene set enrichment analysis using differentially expressed genes between sham and BCAS-operated mice showed activation of oligodendrogenesis pathways along with angiogenic responses. This database of transcriptomic profiles of BCAS-hypoperfusion mice will be useful for future studies to find a therapeutic target for SIVD.

## Introduction

Vascular cognitive impairment and dementia (VCID) syndrome is clinically defined as cognitive decline with evidence of subcortical brain infarction (Erkinjuntti et al., 2000a,b). Subcortical ischemic vascular dementia (SIVD) is the most common subtype of VCID, and patients with SIVD suffer from a vast amount of white matter degeneration due to prolonged cerebral hypoperfusion. White matter damage is a clinically important parameter, as the severity of white matter lesions correlates strongly with the degree of cognitive dysfunction (Pantoni and Garcia, 1997; Medana and Esiri, 2003; Esiri, 2007; Lampe et al., 2019). In SIVD, white matter dysfunction is progressive and is often associated with poor neurological outcome (Roman et al., 2002; Longstreth et al., 2005). Although the number of patients with SIVD is predicted to increase with the aging population, to date there are no established treatments for this pathological condition, partly because of a lack of understanding of the gene expression changes under the conditions of SIVD.

To advance the understanding of SIVD pathology and to find effective approaches for this disease, several animal models have been developed (Tsuchiya et al., 1992; Kurumatani et al., 1998; Lin et al., 2001; Shibata et al., 2004; Yang et al., 2018). Because prolonged cerebral hypoperfusion is a major characteristic that leads to white matter dysfunction in SIVD, the mouse model of prolonged cerebral hypoperfusion by bilateral carotid artery stenosis (BCAS) is considered a well-accepted model for SIVD. Hypoperfused-BCAS mice replicate the pathophysiology of SIVD patients, such as oligodendrocyte/myelin damage and cognitive decline (Ihara and Tomimoto, 2011; Ihara et al., 2014). This model has also been used in various pharmacological studies investigating the treatment of SIVD, which led to the discovery of the potential efficacy of some drugs that are currently approved for other clinical indications (Watanabe et al., 2006; Ueno et al., 2009; Miyamoto et al., 2013a, 2014). Therefore, in this study, we utilized the mouse cerebral hypoperfusion model of SIVD to examine the gene expression changes in the corpus callosum region after cerebral hypoperfusion.

## Materials and Methods

### Animals

All experimental procedures followed NIH guidelines and were approved by the Massachusetts General Hospital Institutional Animal Care and Use Committee. Male C57BL/6J mice were purchased from The Jackson Laboratory and were housed in a specific pathogen-free conditioned 12-h light/dark cycle room with free access to food and water throughout the experiment. A total of 12 male mice (8 weeks old) were used in this study.

### Prolonged Cerebral Hypoperfusion Model by Bilateral Carotid Artery Stenosis (BCAS)

After a week-long habituation period in our animal facility, 12 mice were randomly divided into two groups, for sham operation and for BCAS operation. For the mice in the BCAS group, a microcoil (0.18 mm diameter; Samini, Japan) was applied to the bilateral common carotid arteries for the induction of chronic cerebral hypoperfusion as previously described (Shibata et al., 2004). The sham group received a cervical incision followed by exposure of the bilateral common carotid arteries without microcoil application. Body weight of the mice was measured before operation and 4, 7, 14, 21, and 28 days after operation. No intra-operative or post-surgical complications were observed in this study.

### Novel Object Recognition Test (NORT)

Four weeks after sham or BCAS operation, mice were tested for short-term recognition memory by NORT between 8 and 10 am, as previously described with slight modifications (Wang et al., 2004; Kleschevnikov et al., 2012; Ohtomo et al., 2020). Briefly, mice were placed in a clean empty cage for 10 min. Mice were then exposed to two identical objects in the same cage for 5 min (acquisition period). After an interval of 30 min, mice were then presented with two different objects (one original and one novel object, which were placed in the same position as the objects in the acquisition period) in the same cage for 5 min (retention period). Object recognition was videotaped and scored by the total investigation time either sniffing or touching the object. The performance of short-term recognition memory was described by the ratio of the time spent on the new object to the total time spent on both objects minus 0.5 (e.g., Discrimination index: ranged from −0.5 to 0.5). Experiments and analyses were conducted by an investigator who was blinded to the group allocation.

### Corpus Callosum Sampling

One day after NORT, mice were transcardially perfused with ice-cold 0.9% physiological saline followed by decapitation. Brains were then removed and cooled in ice-cold Hanks’ Balanced Salt Solution for 1 min. After removal of meninges and the choroid plexus, the cerebrum was sliced into five coronal sections using a brain matrix. To minimize inclusion of tissue outside of the corpus callosum., the thicker parts of the corpus callosum from the 2nd and 3rd slices were isolated with direct visualization using a light microscope. Samples of the corpus callosum were put into an RNA free tube and then quickly frozen using liquid nitrogen.

### RNA Extraction

RNA extraction from the corpus callosum samples was performed using QIAzol^®^ (QIAGEN, Germany) following manufacturer’s instructions. Briefly, sonicated tissue was resuspended in 1 ml of ice-cold QIAzol, and 0.2 ml of chloroform was added to the lysate. After mixing by Vortex Mixer, the tube was centrifuged for 15 min at 12,000 *g*. The supernatant was then transferred to another tube, and the same amount of propanol was added. After centrifugation for 10 min at 12,000 *g*, the supernatant was aspirated, and 1 ml of 75% ethanol was added. Finally, the tube was centrifuged for 5 min at 7,500 *g*, followed by suspension with nucleus-free water. The amount and purity of purified RNA was measured by NanoDrop Spectrophotometers. The RNA sample was stored at −80°C before use.

### RNA Sequencing (RNAseq)

Three RNA samples from each group were randomly selected for RNAseq experiments. Library preparation and RNAseq was performed by Genewiz, Inc. (NJ, United States). Libraries for RNAseq were prepared based on the PolyA selection method, and RNAseq was performed by Illumina HiSeq 2 × 150 bp sequencing (single index). The raw data was obtained in FASTQ format, and Kallisto (ver. 0.46.2) was used for quantifying the abundance of transcripts, expressed as transcript per kilobase million (TPM). The bioinformatics analysis was conducted using the R software (ver. 4.0.0). DESeq2 was used for differential expression analysis, and the level with adjusted *p*-value <0.1 was set to filter differential expression genes (DEGs). Metascape was used for the gene set enrichment analysis (Tripathi et al., 2015). The sequence data (FASTQ files) were deposited under the accession #PRJNA727284.

### Statistical Methods

Statistical analysis was conducted by unpaired *t*-test for the NORT data and two-way repeated-measures analysis of variance followed by *post hoc* multiple comparisons test for the body weight data. Differences with *p* < 0.05 were considered statistically significant, and data were expressed as mean ± SD.

## Results

**FIGURE 1:**
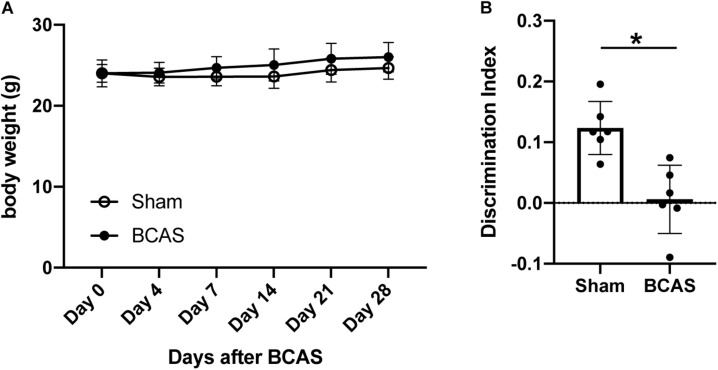
Body weight changes and cognitive function after cerebral hypoperfusion. **(A)** Body weight changes after cerebral hypoperfusion. There was no significant difference between sham-operated and BCAS-hypoperfusion mice. Data are expressed as mean ± SD. *N* = 6 each. **(B)** Cognitive function was assessed by NORT at 4 weeks after sham or BCAS operation. While sham-operated mice accessed the novel object, BCAS-hypoperfusion mice did not show any preference between the novel and familiar objects. Data are expressed as mean ± SD. *N* = 6 each. *p < 0.05.

To prepare a mouse model of SIVD, 2-month-old male C57BL/6J mice were subjected to bilateral common carotid artery stenosis (BCAS) by placing microcoils on both common carotid arteries. There was no significant difference in body weight between sham- and BCAS-operated groups up to 4 weeks after hypoperfusion (Figure 1A). In the novel object recognition test (NORT), BCAS-operated mice showed no preference between familial and novel objects, whereas sham-operated mice spent more time investigating the novel object (Figure 1B), confirming that 4-week cerebral hypoperfusion caused cognitive decline.

**FIGURE 2:**
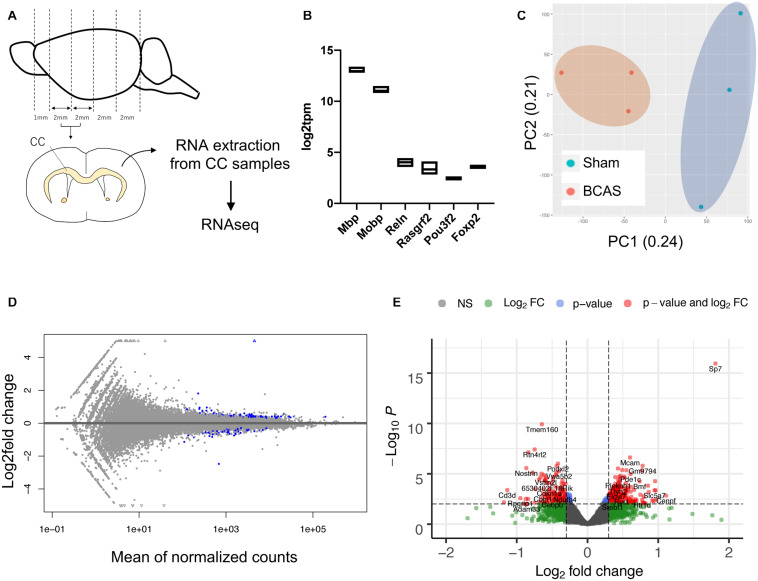
Gene expression changes in mouse corpus callosum after 4-week cerebral hypoperfusion. **(A)** Diagram for corpus callosum preparation. Four weeks after sham or BCAS operation, mice were sacrificed, and the corpus callosum samples were prepared. From each group, three mice were used for the RNAseq studies. CC: corpus callosum. **(B)** Gene expression levels of oligodendrocyte markers (Mbp and Mobp) were much higher than that of cortical neuron markers (Reln for layer I, Rasgrf2 for layer II/III, Pou3f2 for layers II-V, and Foxp2 for layer IV). **(C)** The principal component analysis (PCA) plot. **(D)** The MA plot. Blue dots represent the genes with adjusted *p*-value < 0.1 against the sham group. **(E)** The volcano plot. The red dots represent the genes that showed | log2 fold change| > 0.3 with adjusted *p*-value < 0.1 against the sham group. Please see Supplementary Table 2 for the list of differentially expressed genes between sham- and BCAS-operated mice.

We next isolated RNA samples from the corpus callosum region of mice that have been subjected to BCAS-hypoperfusion for 4 weeks (Figure 2A). The quality of our RNA samples and RNA sequencing was high (Supplementary Table 1), and transcription levels of oligodendrocyte markers (Mbp and Mobp) were significantly higher than the cortical neuron markers (Reln for layer I, Rasgrf2 for layer II/III, Pou3f2 for layers II-V, and Foxp2 for layer IV) (Figure 2B), confirming the purity of our corpus callosum samples. In addition, the principal component analysis (PCA) indicated that the cluster of sham mice data were distinct from the cluster of BCAS mice data (Figure 2C). The MA plot (Figure 2D) and the volcano plot (Figure 2E) revealed that while the gene expression changes caused by 4-week cerebral hypoperfusion were relatively mild, there were several upregulated or downregulated genes in the corpus callosum of hypoperfused mice. The list of differentially expressed genes between sham- and BCAS-operated mice is provided in Supplementary Table 2.

**FIGURE 3:**
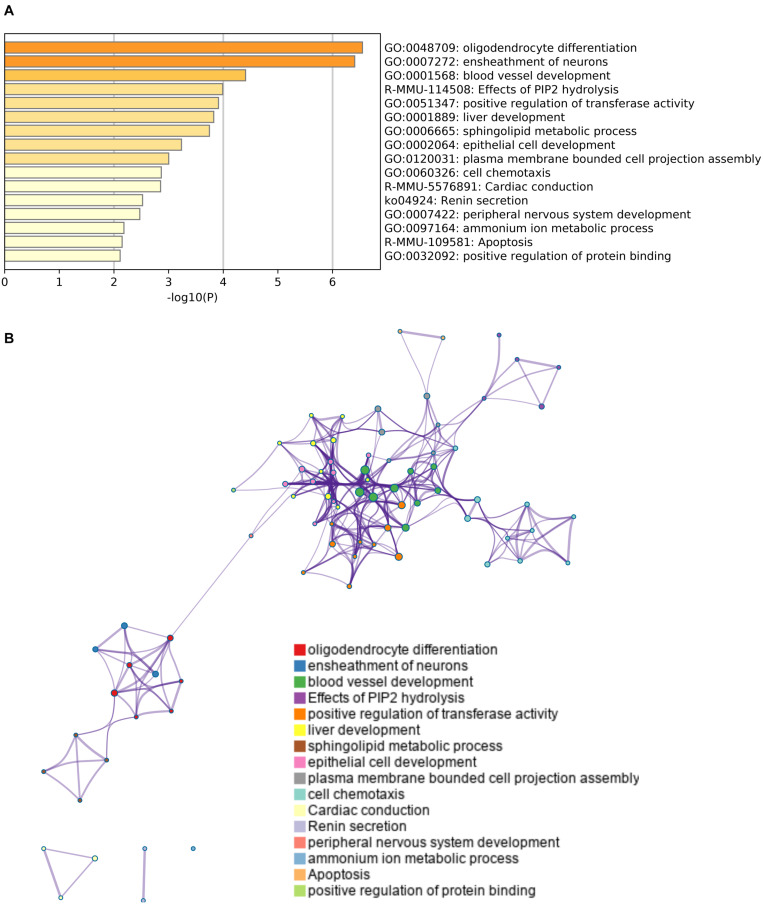
Gene set enrichment analysis of upregulated genes. **(A)** A heatmap of enriched terms across the input genes list. Darker colors indicate smaller *p* values. Upregulated genes were related to the pathways for oligodendrogenesis (GO: 0048709 and GO: 0007272) and angiogenesis (GO: 0001568). Please see Supplementary Table 3 for the list of enriched terms of upregulated genes. **(B)** Metascape enrichment analysis confirms the close relationship between GO: 0048709 (oligodendrocyte differentiation) and GO: 0007272 (ensheathment of neurons). Clustering was made based on similarity (similarity > 0.3).

**FIGURE 4:**
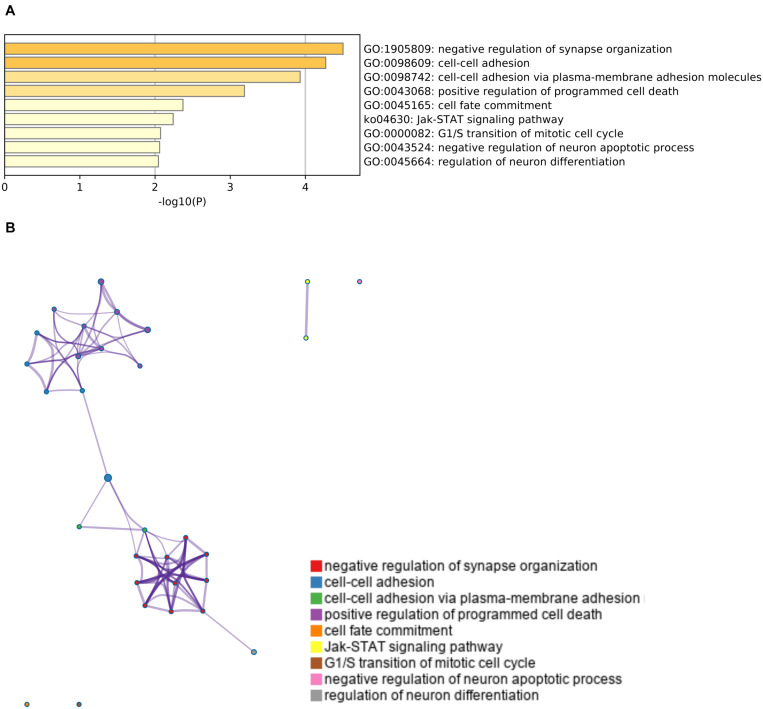
Gene set enrichment analysis of downregulated genes. **(A)** A heatmap of enriched terms across the input genes list. Darker colors indicate smaller *p* values. Downregulated genes were related to the pathways that mediate negative regulation of synapse organization (GO: 1905809) and cell-cell adhesion (GO: 0098609 and GO: 0098742). Please see Supplementary Table 4 for the list of enriched terms of downregulated genes. **(B)** Metascape enrichment analysis. Clustering was made based on similarity (similarity > 0.3).

Finally, we conducted a gene ontology analysis to identify the signaling pathways that were enriched in the upregulated or downregulated genes after cerebral hypoperfusion. For the upregulated genes, pathways that are related to oligodendrocyte/myelin formation and vascular development were highly enriched (Figure 3 and Supplementary Table 3). For the downregulated genes, pathways that are related to the negative regulation of synapse organization and cell-cell adhesion were highly enriched (Figure 4 and Supplementary Table 3).

## Discussion

In this study, we used RNA sequencing analyses to examine transcriptomic changes in the mouse corpus callosum after 4 weeks of cerebral hypoperfusion. Our initial findings suggest that (i) transcriptomic changes in the mouse corpus callosum were relatively mild, (ii) upregulated genes were related to pro-oligodendrogenic and pro-angiogenic pathways, and (iii) downregulated genes were related to cell-cell adhesion pathways. These findings have the potential to lay the groundwork for the research identifying and developing effective therapies for SIVD and other white matter-related diseases.

One major innovation of this study is the study of gene expression in the corpus callosum region using RNAseq. White matter dysfunction is a major feature of many CNS diseases; however; basic research of CNS diseases has mostly focused on the pathological mechanisms of gray matter. This is partly because the volume of white matter is much smaller than that of gray matter in rodents (Zhang and Sejnowski, 2000). However, some rodent models of CNS diseases could be used to examine the pathological mechanisms in cerebral white matter (Arai and Lo, 2009). For example, the BCAS-hypoperfusion model is now well-accepted as a mouse model of SIVD (Ihara and Tomimoto, 2011; Ihara et al., 2014), and our current data support and confirm its utility in the study of cognitive decline along with white matter damage. It is expected that oligodendrogenesis pathways would be activated in the mouse corpus callosum after cerebral hypoperfusion because in young mice, the numbers of oligodendrocyte precursor cells (OPCs) and newly generated oligodendrocytes were reported to be transiently increased as a compensatory response after hypoperfusion (Miyamoto et al., 2013b; Arai, 2020). While no studies have carefully examined the angiogenic responses in the BCAS-hypoperfused mice so far, activation of angiogenic responses had been confirmed in multiple rodent models of white matter damage (Kanazawa et al., 2019; Rust, 2020; Shindo et al., 2021). Interestingly, a microarray study using corpus callosum samples from 3-day cerebral hypoperfusion mice showed an upregulation of angiogenesis-related genes (Reimer et al., 2011). Thus, upregulation of angiogenic pathways would also be expected in the corpus callosum after cerebral hypoperfusion. In addition, our findings that cell-cell adhesion genes were downregulated after cerebral hypoperfusion is consistent with the idea that plasticity of the micro-environment contributes to brain repair/remodeling after injury (Lo, 2008). Furthermore, Rtn4r12 was one of the most significantly downregulated genes in the hypoperfused-BCAS mice. Rtn4r12 encodes Reticulon-4 receptor-like 2 (also known as Nogo-66 Receptor Homolog NgR2), which is a receptor for myelin-associated glycoprotein (MAG) and acts selectively to mediate MAG inhibitory responses (Venkatesh et al., 2005). Taken together, our database of gene expression profiles in the mouse corpus callosum after 4-week cerebral hypoperfusion will be useful to examine the pathological mechanisms of white matter damage/recovery in SIVD and other white matter-related diseases.

Although our study showed that multiple pathways were affected by cerebral hypoperfusion in mouse corpus callosum, it should be noted that the transcriptomic changes after 4-week cerebral hypoperfusion were mild, with only a few genes exhibiting changes of more than 2-fold or less than 0.5-fold. This is partly because the stress of cerebral hypoperfusion by BCAS is prolonged and mild but not acute and severe (Shibata et al., 2004), thus causing a gradual detrimental effect on gene expression in the corpus callosum region. This mild change in gene expression pattern after cerebral hypoperfusion in mice is consistent with previous reports. While our study is the first RNAseq experiment for profiling transcriptomic changes in the corpus callosum after cerebral hypoperfusion, one previous study used microarray analyses with corpus callosum samples of BCAS-hypoperfusion mice and showed that the gene expression changes from 2-week to 6-week after hypoperfusion were mild, matching our current findings (Ohtomo et al., 2018). Based on our findings, future studies are warranted to expand the map of gene expression profiles in the corpus callosum of BCAS-hypoperfused mice with different sets of conditions, such as male vs. female, young vs. old, and early vs. late time points after cerebral hypoperfusion. Because OPC functions display some sex-associated differences (Yasuda et al., 2020) and because aging dampens the compensatory response of OPCs and endothelial progenitor cells (Miyamoto et al., 2013b; Pradillo et al., 2019), understanding how these variables affect transcriptomic profiles of the corpus callosum after hypoperfusion may be a key step in finding novel therapeutic approaches for SIVD and other white matter-related diseases.

This study provides a novel dataset of gene expression profiles in the corpus callosum in BCAS-hypoperfused mice. However, there are several caveats to keep in mind. First, our current study is somewhat preliminary and descriptive, and our finding is not directly related to identification of a therapeutic target for SIVD. Within the differentially expressed gene list from our study, Sp7 showed the most significant difference. Sp7, also called Osterix, is a transcription factor, which plays a role in driving the differentiation of mesenchymal precursor cells into osteoblasts and eventually osteocytes (Sinha and Zhou, 2013). While its role in cerebral white matter is mostly unknown, it was reported that Sp7 is highly expressed in oligodendrocytes (Tabula Muris Consortium, 2018) and would participate in oligodendrocyte maturation (He et al., 2016). Therefore, it is possible that examining the roles of Sp7 in white matter damage and recovery could lead to a novel therapeutic target for SIVD. Second, although we focused on mRNA profiles, there are multiple pseudo-genes in our differentially expressed gene list (Supplementary Table 4), and some of the pseudo-genes, such as Gm24270 and Gm23935, are known to function as miRNA. Future research of changes in the expression of pseudo-genes may enable a deeper understanding of the complex mechanisms of white matter pathology in SIVD. Lastly, the use of “bulk” corpus callosum samples in our study leaves open the possibility that significant changes in gene expression in some cell types may have been missed. It will be useful for future studies to examine gene expression profiles with single cell RNA sequencing to further our understanding of transcriptomic profiles of corpus callosum after cerebral hypoperfusion.

In summary, this preliminary study provides the first database of gene expression profiles in the mouse corpus callosum after 4-week cerebral hypoperfusion. This database may be useful as an initial framework for future investigations of effective therapeutic approaches for SIVD and other white matter-related diseases.

## Data Availability Statement

The data presented in the study are deposited in the NCBI BioProject repository, accession number PRJNA727284.

## Ethics Statement

The animal study was reviewed and approved by the Massachusetts General Hospital Institutional Animal Care and Use Committee.

## Author Contributions

HT, GH, RO, HI, and EM: collection of data. HT, GH, MF, KH, K-HT, EL, and KA: data analysis. KC, JL, MF, KH, K-HT, EL, and KA: manuscript writing. HT, GH, RO, HI, KC, MF, KH, K-HT, EL, and KA: interpretation. MF, KH, K-HT, EL, and KA: conception and design. MF, KH, K-HT, and KA: funding acquisition. HT, GH, RO, HI, KC, EM, JL, MF, KH, K-HT, EL, KA: final approval of manuscript. All authors contributed to the article and approved the submitted version.

## Conflict of Interest

The authors declare that the research was conducted in the absence of any commercial or financial relationships that could be construed as a potential conflict of interest.
